# Utilizing a Cortical Bone Trajectory Pedicle Screw for Lumbar Flexion-Distraction Injury

**DOI:** 10.1155/2018/8185051

**Published:** 2018-06-10

**Authors:** Naohisa Miyakoshi, Shigeto Maekawa, Masakazu Urayama, Yoichi Shimada

**Affiliations:** ^1^Department of Orthopedic Surgery, Akita University Graduate School of Medicine, 1-1-1 Hondo, Akita 010-8543, Japan; ^2^Department of Orthopedic Surgery, Ogachi Central Hospital, 25 Yamada-Yugaoka, Yuzawa 012-0055, Japan

## Abstract

Spinal flexion-distraction injuries (FDIs) are unstable fractures, commonly located at the thoracolumbar junction. Management of FDIs often necessitates the use of posterior instrumentation and fusion, but long-segment instrumentation surgery decreases postoperative spinal mobility and increases the risk of junctional kyphosis and fracture. We report the case of a patient with FDI showing an L2 vertebral fracture, unilateral L2 pedicle fracture, and disruptions of the posterior ligamentous complex between L1 and L2. After open reduction using L1 and L2 pedicle screws with a conventional trajectory on the right side, a cortical bone trajectory (CBT) pedicle screw was used as an osteosynthesis screw for the fractured left pedicle. This procedure enabled successful single-level fusion. Follow-up radiological examination revealed good reduction and complete bone union. To the best of our knowledge, utilizing a CBT technique as an osteosynthesis screw in FDIs has not previously been described.

## 1. Introduction

Spinal flexion-distraction injuries (FDIs) are unstable fractures commonly located at the thoracolumbar junction [1]. This injury pattern was initially classified by Chance [2], who originally described hyperflexion injury to the vertebrae resulting in a disruption of the posterior arch. Classically, the Chance fracture is a pure-bone injury without ligamentous injury comprising a fracture line through the spinous process, laminae, transverse processes, pedicles, and into the vertebral body. However, involvement of soft tissues, such as the posterior ligamentous complex, is very common in spinal FDIs [3]. The classification of FDIs thus now includes monosegmental osseous failure of the posterior tension band extending into the vertebral body, also known as classical Chance fracture, and disruption of the posterior tension band with or without osseous involvement [4]. In the most recent comprehensive classification of thoracolumbar spine injuries proposed by AOSpine, a clinical division of the AO Foundation (Arbeitsgemeinschaft für Osteosynthesefragen), these comprise classification types B1 and B2, respectively [4].

Management of FDIs often necessitates the use of posterior instrumentation and fusion, as conservative management may lead to kyphotic deformities or nonunions [5, 6]. A recent review analyzing the surgical results of FDIs from 13 manuscripts reported long-segment posterior fixation with pedicle screws and rods as the most established surgical treatment in the literature, resulting in sustained improvements in kyphosis, neurological status, and functional back pain assessments [1]. However, long-segment instrumentation surgery decreases postoperative spinal mobility and increases the risk of junctional kyphosis and fracture [7, 8]. Shorter spinal fixation should thus be considered where possible.

Here, we report a case of FDI showing a fracture line through the transverse process and pedicle on one side, and into the vertebral body, combined with disruptions of the posterior ligamentous complex, in which single-level fusion was successfully achieved with the utilization of a cortical bone trajectory (CBT) pedicle screw. The CBT screw technique, with trajectory from medial to lateral and caudal to cranial, was developed by Santoni et al. [9] in 2009 as an alternative strategy to obtain improved pedicle screw fixation in the lumbar spine, and is now commonly used in combination with interbody fusion for the treatment of degenerative lumbar spine diseases [10, 11]. To the best of our knowledge, utilizing a CBT pedicle screw as an osteosynthesis screw in FDIs has not previously been described.

## 2. Case Presentation

A 33-year-old man was involved in an automobile accident and was brought to our hospital by ambulance. He had been in the front passenger seat and had been wearing a three-point seatbelt. He reported severe back pain, but showed no neurological deficit.

**Figure 1:**
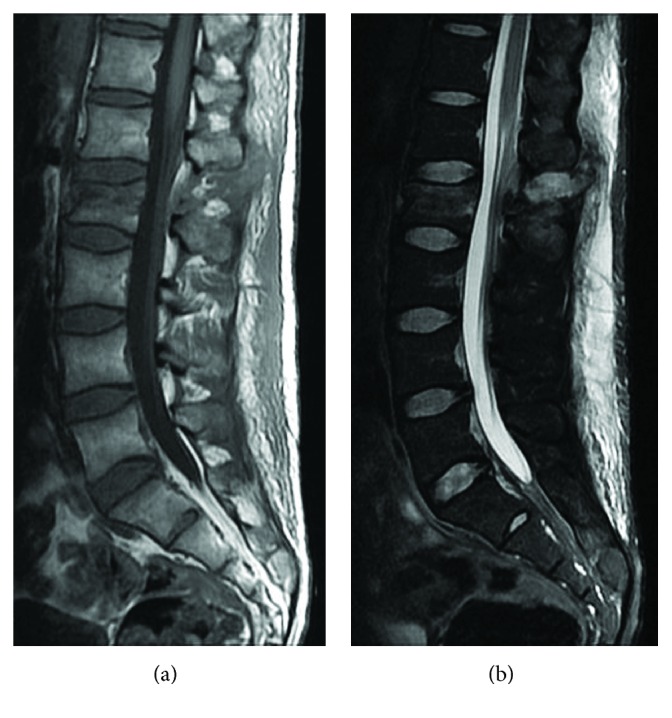
Preoperative magnetic resonance imaging (MRI) of the lumbar spine. (a) Midsagittal T1-weighted and (b) short tau inversion and recovery (STIR) MRI showing the L2 vertebral fracture and disruption of the posterior ligamentous complex between L1 and L2 in combination with extensive subcutaneous hematoma.

Anteroposterior and lateral radiographs of the spine showed an increased gap between the 1st and 2nd lumbar spinous processes and 2nd lumbar vertebral fracture (figures not shown). Magnetic resonance imaging (MRI) of the spine also demonstrated an L2 vertebral fracture and disruptions of the posterior ligamentous complex between L1 and L2, in combination with extensive subcutaneous hematoma (Figure 1).

**Figure 2:**
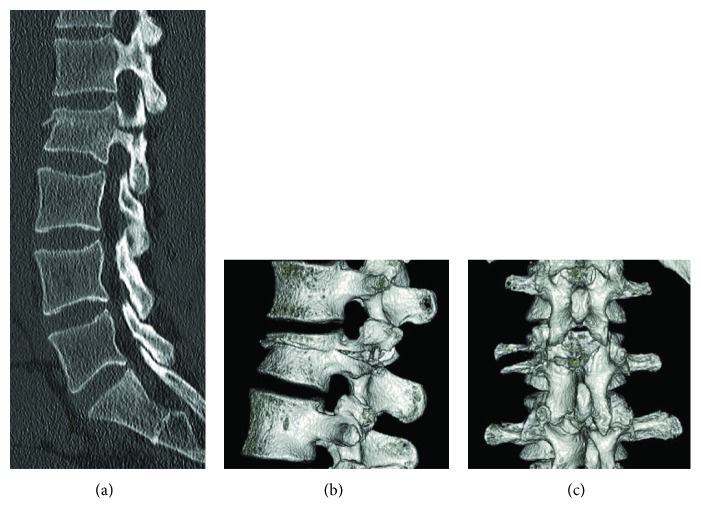
Preoperative computed tomography (CT) of the lumbar spine. (a) Left parasagittal CT showing an L2 fracture involving the vertebral body and extending through the pedicle posteriorly. (b) 3D-CTs from the left lateral view and (c) posterior view show that the fracture consists of horizontal splitting from the vertebra, through the left pedicle and transverse process, and reaching to the mid-upper neural arch.

Computed tomography (CT) of the spine in the sagittal orientation and 3-dimensional (3D) CT further revealed the involvement of the superior end plate of the L2 vertebra, comprising horizontal splitting from the left pedicle, through the left transverse process, and reaching the center of the neural arch (Figure 2). The right-sided L2 pedicle was intact.

**Figure 3:**
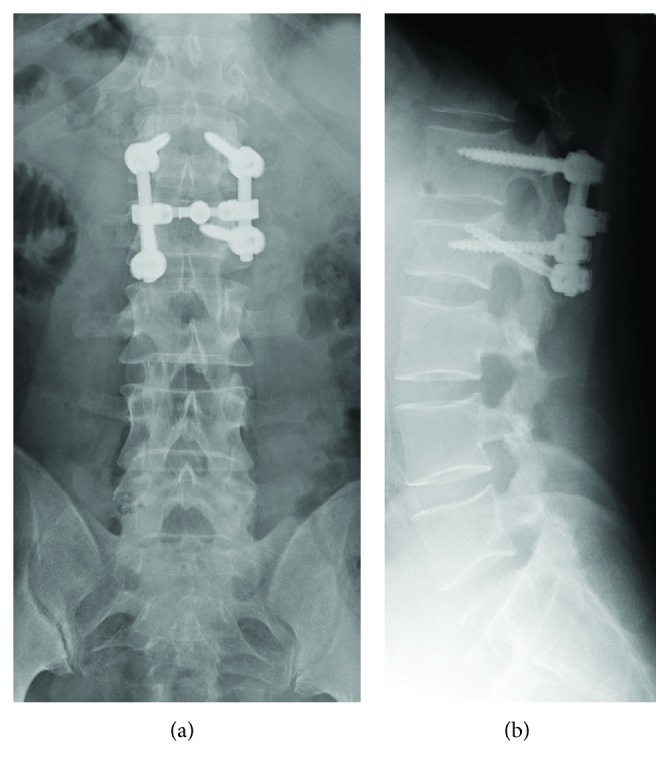
Postoperative plain X-rays of the lumbar spine. (a) Anteroposterior and (b) lateral radiographs show an L1-L2 single-level instrumented fusion using a CBT pedicle screw for L2 on the left side.

After checking the general condition of the patient and excluding intra-abdominal injury by enhanced CT and ultrasonography, the patient underwent L1-L2 single-level instrumented fusion using a posterior approach. Initially, monoaxial pedicle screws with conventional trajectory were placed at L1 and L2 pedicles on the right side (intact pedicle side). A rod slightly bent in lordosis was then introduced and connected with these pedicle screws with a compression force applied between screws. This procedure achieved reduction and the fracture gap at the left L2 pedicle and lamina was completely closed. Polyaxial pedicle screws were used on the left side. A pedicle screw with a conventional trajectory was placed at the left L1 pedicle. A CBT pedicle screw was then inserted through the fractured L2 pedicle under fluoroscopy. This CBT screw was used as an alternative to an osteosynthesis screw. A rod was introduced on the left side, bilateral facet fusion with local bones obtained from the lower one-third of the L1 spinous process was performed, and the wound was closed. Although the merits of cross connectors for CBT screws remain unclear, we applied the connector in this case because connecting bilateral pedicle screws along conventional trajectories has been reported to increase the pullout strength of these screws [12]. Postoperative X-rays showed good reduction by this single-level fixation (Figure 3).

**Figure 4:**
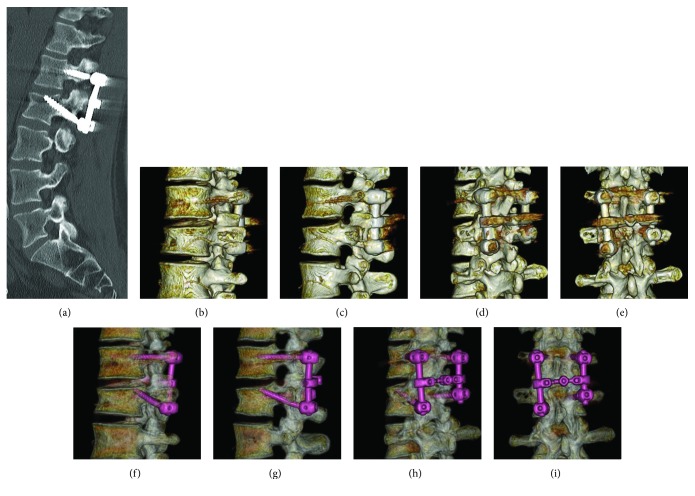
Postoperative computed tomography (CT) of the lumbar spine. (a) Left parasagittal CT obtained 6 months after surgery showing good reduction and fracture healing of the L2 vertebral body and pedicle, with proper CBT screw placement. (b, f) 3D-CTs obtained 1 year after surgery from the left anterolateral view, (c, g) left lateral view, (d, h) left posterolateral view, and (e, i) posterior view showing the screw trajectories in 3D view and complete bone union of the fracture, including the L2 vertebral body, pedicle, and transverse process. (f–i) Bone density was reduced to observe screw trajectories.

The postoperative period was uneventful. Although rigid fixation was obtained with this procedure, a thoracolumbosacral orthosis (TLSO) was applied for 6 weeks, since this case was our first experience. Physical activities were not restricted with the TLSO. Sagittal CT and 3D-CT obtained at 6 months and 1 year postoperatively showed proper trajectory of the CBT pedicle screw and complete bone union (Figure 4).

## 3. Discussion

The most common treatment procedure for spinal FDIs is surgical reduction and rigid instrumented fixation, and postoperative TLSO is usually unnecessary. Among FDIs, classical Chance fractures (pure-bone fractures) may be treated conservatively by closed reduction and immobilization in a TLSO or extension cast [13]. Pure-bone fracture can also be treated by instrumentation without fusion. However, FDIs with disruptions of the posterior ligamentous complex should be treated surgically because ligamentous insufficiency induces progressive kyphosis even after the bony fracture has healed. In the present case, since the posterior ligamentous complex between L1 and L2 was disrupted, surgical fixation was performed. Single-level instrumented fusion was applied in this case, but the conventional pedicle screw technique could not be applied for the left L2 pedicle because of the fracture. A two-level fusion (L1–L3) would usually be indicated for such cases.

In this case, we used a CBT pedicle screw as an alternative to an osteosynthesis screw for the L2 transverse pedicle fracture, because the trajectory of this technique can pass through the plane of the fracture. In addition, the CBT pedicle screw could help stabilize the affected L1-L2 segment, and this technique thus enabled single-level fusion. However, when we use a CBT pedicle screw as an osteosynthesis screw, a reduction of the fracture is necessary and the gap must be closed before screw insertion. We thus suggest the importance of two more specific points in this case to complete a single-level fusion. First, since the right L2 pedicle was intact, we could use a pedicle screw with a conventional trajectory for this pedicle. This could help in the reduction of the fracture. Second, we used monoaxial screws for L1 and L2 pedicles. The pedicle screw-rod construct is very important for spinal correction. A monoaxial screw creates a better screw-rod construct than polyaxial screws. Because the relationship between the monoaxial screw and rod is fixed at 90° when connected, the correction angle is faithful to the angle of rod bending. In this case, the use of monoaxial pedicle screws for the right side might have facilitated reduction. Thus, if the gap can be closed at the transversely fractured pedicle in a case of FDI, utilizing a CBT pedicle screw may offer a good option for bone union and minimizing fusion levels.

Temporary use of percutaneous pedicle screw (PPS) placement without fusion can be a treatment option for FDIs, particularly for a pure Chance fracture (which has no ligamentous injury), because removal of the instrumentation after fracture healing could preserve motion segments. One study showed that PPS fixation appears to offer similar efficacy in the treatment of FDI and allows for reduced blood loss and tissue damage compared with open surgical techniques [3]. However, posterior ligamentous injury cannot be completely healed with PPS, so correction loss after removal of the instrument would be an issue of concern. Ligaments sometimes heal naturally, but unlike bone, the preinjury structure, organization, and biomechanical properties are not completely restored, because of the development of scar tissue [14].

Alternatively, two-level monoaxial instrumentation combined with a one-level fusion of the ligamentous injured level with implant removal after bony healing can also represent a good treatment option. However, this option requires longer surgical exposure compared to our procedure, and the patient should undergo surgery twice (initial instrumentation surgery and implant removal surgery). The present procedure requires only a one-time surgery with a shorter wound exposure compared to that option. In our procedure, the CBT technique can only be used in one of the 4 screws, which might limit the indications to specific cases as in the present case. However, when indicated, utilizing a CBT screw could provide a significant advantage compared to other alternative procedures.

## 4. Conclusion

This report presented the case of a surgically treated patient with FDI who showed an L2 vertebral fracture and unilateral L2 pedicle and transverse process fracture, combined with disruptions of the posterior ligamentous complex between L1 and L2. After open reduction, a CBT pedicle screw was utilized as an osteosynthesis screw for the L2 pedicle fracture. This procedure enabled successful single-level fusion. To the best of our knowledge, utilizing a CBT screw as an osteosynthesis screw in FDI has not previously been described.
